# gMISpy: integration of complex regulatory networks and genome scale metabolic models

**DOI:** 10.1093/bioinformatics/btag180

**Published:** 2026-04-08

**Authors:** Carlos J Rodriguez-Flores, Naroa Barrena, Loïc Paulevé, Francisco J Planes

**Affiliations:** Tecnun School of Engineering, University of Navarra, San Sebastián 20018, Spain; Tecnun School of Engineering, University of Navarra, San Sebastián 20018, Spain; Univ. Bordeaux, CNRS, Bordeaux INP, LaBRI, UMR 5800, Talence F-33400, France; Tecnun School of Engineering, University of Navarra, San Sebastián 20018, Spain; Biomedical Engineering Center, University of Navarra, Campus Universitario, Pamplona, Navarra 31009, Spain; Instituto de Ciencia de los Datos e Inteligencia Artificial (DATAI), University of Navarra, Campus Universitario, Pamplona 31080, Spain

## Abstract

**Motivation:**

Genome-scale metabolic models lack explicit regulatory mechanisms, limiting their predictive accuracy for genetic interventions. Current methods for computing genetic Minimal Cut Sets either ignore regulatory networks entirely or use simplified acyclic representations that cannot capture regulatory feedback loops, ubiquitous features critical in cellular modeling.

**Results:**

We developed gMISpy, a Python package that that enables efficient computation of genetic Minimal Intervention Sets (gMISs) in integrated genome-scale metabolic and regulatory networks. gMISpy incorporates cyclic regulatory logic into our previous computational framework using layered Boolean networks and BoNesis framework, resulting in a more accurate modeling of how regulatory interactions affect metabolic genes. Benchmarking across four different regulatory networks with Human-GEM showed consistent improvements in prediction accuracy, with Matthews correlation coefficient gains ranging from 2.50% to 14.42%. Validation against cancer data from DepMap and Project Score confirmed that cyclic integration reduces false positives and better captures biological vulnerabilities compared to acyclic approaches.

**Availability and implementation:**

https://github.com/PlanesLab/cyclic-gMISpy

## 1 Introduction

The characterization of biological systems is challenged by the vast number of molecular interactions that sustain cellular function. Genome-scale metabolic models (GEMs) have emerged as a cornerstone of systems biology, offering ever-improving representations of metabolism and its genetic underpinnings. By integrating genomic, biochemical, and physiological data, GEMs provide a powerful framework for holistic analyses of cellular processes (Gu *et al.* 2019).

A major focus in GEM analysis is the identification of minimal intervention strategies—combinations of gene or reaction modifications that induce desired phenotypic outcomes, such as cell death or metabolic rerouting. The concept of Minimal Cut Sets (MCSs) has proven influential in this domain: MCSs are irreducible sets of reactions whose removal blocks specific metabolic functions (Klamt and Gilles 2004, Klamt *et al.* 2025). Extending this idea to the genetic level led to the concept of genetic Minimal Cut Sets (gMCSs), which identify minimal sets of gene knockouts required to disrupt a metabolic task (Apaolaza *et al.* 2017,^ 2019^).

Since the original introduction of gMCS computation, several key methodological advances have improved its scope and efficiency. In a recent work (Rodriguez-Flores *et al*. 2024), we revised and enhanced the performance of gMCS calculations by providing an efficient implementation in Python, called gMCSpy. In addition, Barrena *et al.* (2023) integrated acyclic regulatory information by extending Gene–protein–reaction (GPR) rules, which define Boolean equations that encode the mapping between genes, their protein products, and associated metabolic reactions. More recently, the optimization framework was reformulated to enable simultaneous computation of knock-in and knockout interventions, presenting the concept of genetic of Minimal Intervention Sets (gMISs). However, the main limitation of these works is that acyclic regulatory logic does not fully capture feedback loops or cyclic behaviors, ubiquitous features in biological networks that are critical for accurate modeling.

Here, we introduce gMISpy, a novel open-source Python package that enables efficient computation of genetic Minimal Intervention Sets (gMISs) in integrated genome-scale metabolic and regulatory models. Crucially, we incorporate cyclic regulatory logic by integrating our previous modeling framework with Bonesis (Chevalier *et al.* 2024), a computational tool that enables dynamic analysis of cyclic Boolean Networks, resulting in a more accurate modeling of how regulatory interactions can affect metabolic genes. We demonstrate that our method enables precise identification of lethal genetic interactions in large-scale human network models. Finally, we benchmark gMISpy across multiple regulatory networks and evaluate its predictive power using large-scale CRISPR genetic screens from DepMap (Arafeh *et al.* 2025) and Project Score (Behan *et al.* 2019).

## 2 Methods

### 2.1 Input data for gMISpy

**Figure 1:**
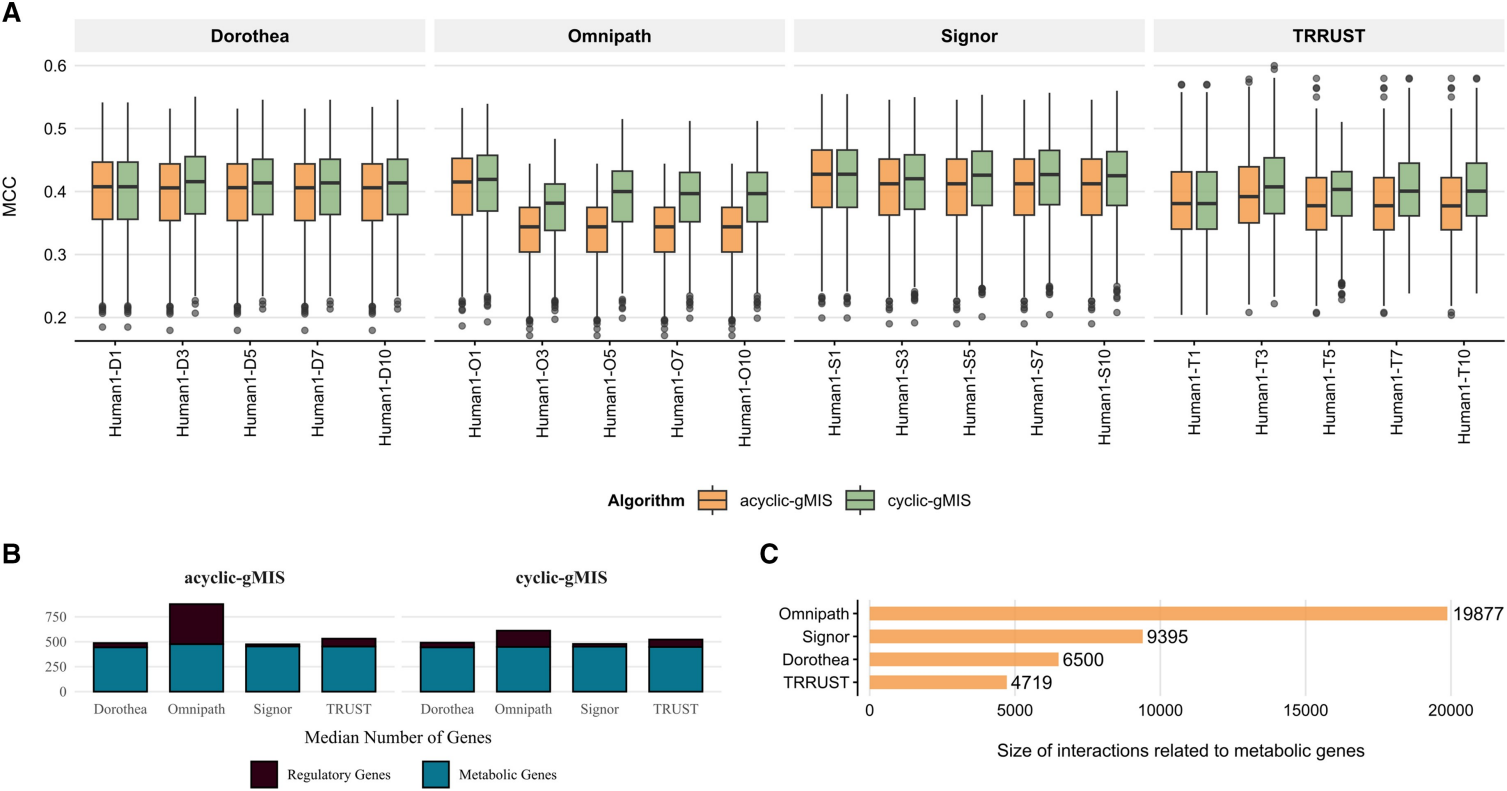
Performance comparison and gene composition across network configurations and algorithms. (A) Matthews correlation coefficient (MCC), grouped by network configuration and algorithm, based on DepMap data. Network configurations include GEM Humanv1.19 combined with multiple networks: Dorothea, Omnipath, Signor, and TRRUST. Each configuration is subdivided into two groups: acyclic gMIS and cyclic gMIS, with facets according to the number of layers used in the analysis (1,3,5,7,10). (B) The median gene space composition in gMISs computed by each algorithm. (C) Annotated interactions of regulatory genes and metabolic genes in different models.

The principal innovation of gMISpy lies on a dedicated data structure, called GDict, which enables an efficient calculation of genetic minimal intervention sets (gMISs) from integrated metabolic and regulatory networks, going beyond the limitation of linear acyclic pathways assumed in our previous works. GDict stores matrices G and F, which define the gene knockout and knock-in constraints required in our underlying mixed-integer linear programming (MILP) model (Fig. 1, available as supplementary data at *Bioinformatics* online).

GDict is derived from extended Gene–Protein–Reaction (eGPR) rules, which integrate GPR rules from GEMs and regulatory networks into a single coherent Boolean Network (BN). This integration employs a layered regulatory expansion, similar to that of Barrena *et al.* (2023), but enabling cyclic behaviors. In this framework, direct regulatory interactions are designated as layer 1 (e.g. gene B regulates gene A), while upstream regulators (e.g. gene C regulating gene B) are assigned to layer 2, and so on. The number of layers is variable and depends on the depth of regulatory influence desired. This iterative process may introduce inconsistencies and disable the network to reach an active state of a reaction. To prevent this, we confirm after each expansion the existence of an attractor where the target reaction is reachable.

### 2.2 BoNesis integration for GDict computation

GDict construction relies on logic programming techniques, leveraging the BoNesis framework (Chevalier *et al.* 2024), which enables dynamic analysis of Boolean Networks using Answer Set Programming (ASP). For each reaction, BoNesis analyzes the attractors of the associated BN, i.e. stable states or recurring dynamic patterns to which the network converges over time, and identifies the minimal set of gene interventions required for its inactivation.

Our implementation uses BoNesis’s marker reprogramming algorithm (Paulevé 2023), which identifies the perturbations required to force the Boolean Network’s attractors to satisfy a user-defined marker, in our case, rendering the target reaction inactive. To accomplish this task, each reaction and its associated eGPR are first encoded as a Boolean network, and then all minimal subsets of gene perturbations leading to the inactivation of the target reaction are systematically identified by BoNesis.

To further enhance the efficiency of GDict construction, each reaction is processed in parallel, allowing to greatly reduce computation times. Once all reactions have been processed, the resulting GDict structure serves as the input for the MILP-based gMIS computation, following the methodology described in Barrena *et al.* (2026) (see Supplementary Material, available as supplementary data at *Bioinformatics* online).

### 2.3 Comparative analysis using DepMap and project score data

We benchmarked gMISpy-acyclic (gMIS calculated from acyclic integration of regulatory networks), and gMISpy-cyclic (gMIS from cyclic integration of regulatory networks). The comparative analysis tested four regulatory networks (Dorothea (Garcia-Alonso *et al*. 2019), TRRUST (Han *et al.* 2018), Signor (Surdo 2023), Omnipath (Türei *et al*. 2016) with the latest Human-GEM (v1.19) (Wang *et al.* 2024). gMISpy-acyclic, implemented in Barrena *et al*. (2026) served as the baseline, based on our assumption that adding cyclic regulatory information should improve predicted gMIS accuracy.

Large-scale validation was performed using DepMap and Project Score data, which provide expression profiles and knockdown experiments across cell lines. gMISs can include either gene knock-outs ($g_{i}^{-}$) or gene knock-ins ($g_{i}^{+}$). As done in Barrena *et al.* (2023), we classify a gene $g_{i}^{-}$ as potentially essential in a particular sample if it is the unique highly expressed gene among the specific subset of gene knock-outs in a particular gMIS and, when applicable, the subset of genes to be knocked-in in that gMIS are highly expressed. For instance, in the case of {*g_1_^−^; g_2_^−^*}, in cell lines where $g_{1}$ is not expressed, knocking down $g_{2}$ should result in greatly reduced cell viability (DepMap score ≤ −0.6, Project Score > 0). For mixed interventions, such as like {*g_3_^−^; g_4_^+^*} knocking down $g_{3}$ in cell lines expressing $g_{4}$ should produce scores below −0.6. One limitation of this approach is that neither DepMap nor Project Score contains knock-in experiments, preventing evaluation of gene activation effects on cell viability. Additionally, only single perturbations have been performed, so multiple perturbation hypotheses cannot be directly evaluated.

Once we have identified the potential essential genes in a sample, we need to verify that when they are knocked-out or knocked-in, respectively, the rest of the genes in the gMIS of interest do not become active in the case of gene knock-outs or inactive in the case of gene knock-ins by an adaptation mechanism. This was done efficiently with BoNesis by including an additional postprocessing step of computed gMISs. Specifically, we assessed every single gene knockout involved in gMISs and validated that valid attractor configurations in the Boolean Network can be reached. For instance, in the above example, it is checked that $g_{2}$ does not become active or $g_{4}$ inactive upon the knockout of $g_{1}$ and $g_{3}$, respectively. If no such configuration exists—indicating the network has readapted—the gMIS is flagged and associated essential genes discarded. This postprocessing step significantly reduces false positives. A more detailed explanation can be found in Supplementary Material, available as supplementary data at *Bioinformatics* online and Figs 2 and 3, available as supplementary data at *Bioinformatics* online.

## 3 Results

### 3.1 Benchmark of regulatory network integration methods

The integration of regulatory networks into GEMs resulted in explicit performance differences among the tested computational approaches. For DepMap data, gMISpy-cyclic consistently exhibited enhanced performance as network complexity increased (layers 1–10), as demonstrated by rising trends in Matthew’s correlation coefficient (MCC) metrics across multiple regulatory networks (Figure 1A). In contrast, the acyclic integration approach (gMCSpy-acyclic) displayed static performance profiles with minimal responsiveness to increasing network complexity (Figure 1A). Full details can be found in Figs 4–8, available as supplementary data at *Bioinformatics* online. Similar results were observed for Project Score data (Fig. 9, available as supplementary data at *Bioinformatics* online).

Notably, this improvement in predictive accuracy with cycle integration was observed even when the number of included genes was comparable to or only modestly greater than that of the acyclic approach. For example, in the Signor network the median gene space composition of the computed gMIS is very similar, namely gMISpy-cyclic incorporated 28 regulatory and 451 metabolic genes, while gMISpy-acyclic involved 21 regulatory and 454 metabolic genes (Fig. 1B)—yet the gMISpy-cyclic still achieved higher MCC values (Fig. 1A).

These results underscore that the primary advantage of cycle integration lies not in simply expanding the gene set, but in its capacity to more effectively model complex regulatory feedback and interactions—yielding superior prediction quality as measured by MCC. While the exact distribution of regulatory and metabolic genes varied between networks and methods (Fig. 1C), the observed improvements highlight the central importance of integrated network logic over absolute gene counts. Note here that the increase in accuracy of gMISpy-cyclic over gMISpy-acyclic brings about a slight and acceptable increment in computation time in most cases (Fig. 10, available as supplementary data at *Bioinformatics* online). In addition, the scaling of gMISpy-cyclic to more complex scenarios in terms of number of nodes guarantee its feasibility (Fig. 11, available as supplementary data at *Bioinformatics* online).

### 3.2 gMISpy statistical analysis

To robustly quantify the impact of regulatory network integration on predictive performance, we constructed a linear mixed-effects model using MCC as the dependent variable. MCC was selected because it encompasses all aspects of binary classification (TP, FP, TN, FN), thus providing a balanced measure of overall prediction quality. The model compared the effect of both cyclic and acyclic integration strategies with separate estimates for each regulatory network and their interactions.

This analysis revealed that gMISpy-cyclic consistently yielded statistically significant improvements over gMISpy-acyclic approach for all networks examined. Table 1 summarizes the results for DepMap data. The same analysis for Project Score data can be found in Table 1, available as supplementary data at *Bioinformatics* online.

**Table 1:** Summary of DepMap comparison between gMISpy-acyclic and gMISpy-cyclic.^a^

Network	acyclic-gMISpy mean	cyclic- gMISpy mean	Gain (Δ)	*P*-value	% Gain
Human1-Omnipath	0.336	0.385	0.048	<0.001	14.42
Human1-Dorothea	0.396	0.406	0.010	<0.001	2.50
Human1-Signor	0.403	0.416	0.013	<0.001	3.12
Human1-TRRUST	0.383	0.403	0.020	<0.001	5.17

a Mean MCC value across samples for gMISpy-acyclic and gMISpy-cyclic, absolute and relative gain of cylic-gMISpy over acylic-gMISpy and t-test *P*-value.

As shown in Table 1, integrating the TRRUST network with cycles produced an absolute effect size of 0.020 (SE = 0.0007, p < 0.001), corresponding to a relative improvement of 5.17% over the acyclic baseline. Cyclic integration with Signor resulted in a gain of 0.013 (SE = 0.0007, *P* < 0.001), representing a 3.12% improvement. Similarly, Dorothea showed a modest but significant gain of 0.010 (SE = 0.0007, *P* < 0.001), corresponding to 2.50% enhancement.

The most substantial performance improvement was observed with the Omnipath network, which demonstrated a gain of 0.048 (SE = 0.0007, *P* < 0.001), yielding the highest relative improvement of 14.42%. This behavior can be explained by the fact that Omnipath has both (i) a high percentage of GPRs containing cycles (>80% at higher regulatory layers) and (ii) a dramatically larger number of nodes involved in cycles than the rest of regulatory networks, reaching up to 10-fold increase at higher regulatory layer (Fig. 12, available as supplementary data at *Bioinformatics* online). As a result, a correct modeling of cycles, as done by gMISpy-cyclic, translates into a greater impact on overall performance.

The consistent pattern of statistically significant improvements (*P* < 0.001) across all regulatory networks supports the robustness of the cyclic integration approach for enhancing algorithmic performance in regulatory network analysis.

## 4 Discussion

The central finding of this work is that incorporating cyclic regulation networks into GEMs substantially improves the prediction of gene intervention strategies. Across all four regulatory networks tested, cyclic integration consistently outperformed acyclic approaches, with improvements in MCC scores ranging from 2.50% to 14.42% in DepMap and from 2.13% to 9.74% in Project Score. This consistent pattern suggests that regulatory feedback loops are not merely computational complications to be avoided, but essential features that must be modeled to capture cellular behavior accurately.

The performance gains observed in Table 1 cannot be explained by simply including more regulatory genes. For instance, in the Signor network, cyclic integration achieved superior results despite incorporating fewer total genes than the acyclic approach (479 vs 475). This finding supports our hypothesis that network topology—specifically the presence of feedback loops—is more important than gene count for accurate predictions.

The particularly strong improvement of OmniPath likely reflects its comprehensive coverage of regulatory interactions, which when modeled with cycles, captures the complex feedback mechanisms that govern cellular responses. Consistent with this interpretation, Omnipath exhibits a substantially higher average number of nodes participating in cycles across GPRs compared with other regulatory networks. Notably, while OmniPath still does not achieve the highest absolute performance, the cyclic approach brings it much closer to the rest of the models.

This illustrates that in large resources, such as Omnipath, the presence of cycles is particularly relevant. Under these conditions, gMISpy-cyclic displays a robust outcome, compensating for the lack of quality of regulatory networks, particularly at higher layers. This was further confirmed with Signor, which provides confidence thresholds for regulatory interactions; across three different confidence thresholds, we observed similar performance (Fig. 13, available as supplementary data at *Bioinformatics* online).

The integration of BoNesis for Boolean network analysis enabled us to handle these cyclic regulatory relationships systematically. Our layered expansion approach prevents contradictory information from corrupting the model while maximizing the use of available regulatory data. Furthermore, our postprocessing validation step—which verifies that predicted interventions correspond to valid network states—proved essential for reducing false positives when validated against DepMap and Project Score experimental data.

Our validation approach is centered on only one type of intervention experiments. DepMap and Project Score lack gene activation assays, preventing us from fully evaluating interventions that require gene upregulation—a key capability of our method. Additionally, these large-scale knockout screens only provide single perturbation data, so we cannot directly validate our combinatorial intervention predictions. These limitations suggest that while our improved predictions are promising, more comprehensive experimental validation is needed to fully assess the capabilities of our approach.

Looking forward, our framework could be extended to: (i) capture bidirectional interactions between regulatory genes and metabolic genes and (ii) go beyond metabolic networks and include other cellular processes, potentially revealing intervention strategies that exploit vulnerabilities across multiple biological systems. More immediately, designing regulatory networks centered in cyclic regulation could improve our results drastically. This work represents a meaningful step toward more biologically realistic computational models that better capture the complex regulatory dynamics governing cellular behavior.

## Supplementary Material

btag180_Supplementary_Data

## Data Availability

The data underlying this article are publicly available in cyclic-gMISpy, at https://doi.org/10.1093/bioinformatics/btae318
