# Supplementary material for: gMISpy: integration of complex regulatory networks and genome scale metabolic models
Source: Bioinformatics. 2026 Apr 8;42(4):btag180. doi: 10.1093/bioinformatics/btag180 (PMC13125755; doi:10.1093/bioinformatics/btag180)
Supplement: btag180_Supplementary_Data [file btag180_supplementary_data.docx]

Supplementary Material

gMISpy: Integration of Complex Regulatory Networks and Genome Scale Metabolic Models

Carlos Javier Rodriguez^1^, Naroa Barrena^1^, Loïc Paulevé^2^, Francisco J. Planes^1,3,4*^

^1^University of Navarra, Tecnun School of Engineering, Manuel de Lardizábal 13, 20018 San Sebastián, Spain.

^2^Univ. Bordeaux, CNRS, Bordeaux INP, LaBRI, UMR 5800, F-33400 Talence, France.

^3^University of Navarra, Biomedical Engineering Center, Campus Universitario 31009 Pamplona, Navarra, Spain.

^4^University of Navarra, Instituto de Ciencia de los Datos e Inteligencia Artificial (DATAI), Campus Universitario, 31080, Pamplona, Spain

*^*^Corresponding author:* [*fplanes@tecnun.es*](mailto:fplanes@ceit.es)

**Supplementary Methods**

**MILP formulation for the calculation of gMISs**

In this section, we present a brief description of the mixed integer linear programming (MILP) model, previously developed in different works of our group (Apaolaza et al., 2017, 2019; Barrena et al., 2023, 2025), which allows us to search for genetic minimal intervention sets (gMISs) in genome-scale metabolic models (GEMs). As detailed in the next section, the main innovation in this work lies in the calculation of matrix G in extended GPR rules involving cyclic interactions. The characterization of matrix G is defined in a later subsection.

For a particular GEM, the full set of reactions is typically represented with the stoichiometric matrix, here denoted as $S$, of dimensions $m\times n$, where $m$ and $n$ are the total number of metabolites and reactions, respectively. The values of each column represent the stoichiometric coefficients of the different metabolites in a particular reaction. Products and substrates in a reaction take positive and negative coefficients, respectively. Each reaction can carry a flux $r$; however, we do no not allow negative fluxes and, thus, each reversible reaction must be split into two irreversible reactions:

$r \geq0$ (Eq.1)

Considering the steady state and the mass balance equation, we can establish that consumption and production fluxes must be equal to zero:

$S\cdot r=0$ (Eq.2)

Next, we define the target metabolic task that we aim to block, typically the biomass reaction. Thus, we force a positive flux through this target task:

$t^{T}\cdot r \geq t^{*}$ (Eq.3)

where $t^{T}$represents a row vector in which all its elements are zero except for the reactions implied in the task; and $t^{*}$ is a positive constant.

Eq. 4 defines the gene intervention (gene knockouts or knock-ins) constraints. As detailed in the next section, each row in G specifies the subset of reactions blocked as a result of a minimal subset of gene interventions, stored in *F* matrix.

$G\cdot r \leq0$ *(Eq.4)*

Eqs. 1-4 define the primal linear programming problem. Due to the inconsistency between Eq.3 and Eq.4, the primal problem is infeasible. In that situation, its associated dual problem defines an unbounded polyhedral cone, whose extreme rays represent minimal subsets of constraints in the primal problem that are inconsistent and lead to the underlying infeasibility. This is closely related with the concept of gMISs, which are defined by Eq.3 and minimal subsets of gene intervention constraints in Eq.4. Accordingly, gMISs can be calculated using the following MILP:

$minimize\sum_{i=1}^{i=l} d_{i}\cdot z_{i}$ (Eq.5)

$$s.t.$$

$N\cdot\left( \begin{matrix} u \\ v \\ w \end{matrix} \right)=\left[ S^{T} G^{T}-t \right]\cdot\left( \begin{matrix} u \\ v \\ w \end{matrix} \right)\geq0$ (Eq.6)

$\alpha\cdot z\leq v\leq M\cdot z$ (Eq.7)

$r^{*}\cdot w\leq-c, c>0$ (Eq.8)

$z_{\delta}\geq z_{\beta} \forall\left( \delta, \beta\right)| F\left( \beta\right)\supset F\left( \delta\right)$ (Eq.9)

$\sum_{i=1}^{i=l} z_{i}^{j}z_{i}\leq\sum_{i=1}^{i=l} z_{i}^{j}-1$ (Eq.10)

$v\geq0;w\geq0$ (Eq.11)

$u\in R^{m}, v\in R^{l}, w\in R, z \in B^{l}$ (Eq.12)

where $u$, $v$, and $w$ denote dual variables linked to the mass balance equation, gene intervention constraints, and the target (metabolic task) constraint, respectively. Eq. 6 defines the constraints associated to the dual problem. In addition, $z$ variables are binary variables associated with $v$ variables through Eq.7, which guarantees that if $z=1$ then $v>\alpha$ or if $z=$ 0 then $v=0$. Eq. 8 forces $w$ to be non-zero, incorporating the target constraint, Eq.3, into the infeasible primal problem. Eq.9 addresses the dependencies between dual variables $v$, which may yield non-minimal solutions. Finally, Eq.10 eliminates previous solutions ($z^{j}$) from the solution space and enables the enumeration of gMISs in increasing number of gene interventions. Eqs. 11-12 define the nature of variables used in our MILP. The objective function, Eq.5, is discussed below.

**From GPRs to validated gMIS**

The MILP model defined above, Eqs. (5)-(12), requires the prior definition of matrix G, which serves as the foundational component for constructing gene interventions constraints as specified in Eq 4. Matrix G establishes the relationship between minimal gene intervention subsets and their corresponding disabled reaction groups, where each row represents a specific subset of gene interventions that collectively inactivates a defined subgroup of reactions.

The *gMISpy* framework implements matrix G through a Python dictionary data structure, designated in the main text as *GDict*. This dictionary architecture employs a key-value pair system where keys represent unique gene subsets and values contain lists of associated reactions. This data structure provides computational efficiency while maintaining the necessary mapping relationships between gene knockouts/knock-ins and reaction deactivation patterns.

The construction of *GDict* requires the systematic identification of minimal gene intervention sets to independently block each reaction in the GEM. This is done as follows. First, a Boolean network that represents the logical relationships within each metabolic GPR is constructed (Supplementary Figure 1A). Subsequently, regulatory layers are incorporated into the Boolean network based on predetermined layer specifications, leading to expanded Boolean networks (Supplementary Figure 1B-C), in our previous works denoted as extended GPRs (*eGPRs*). Finally, we employ *Bonesis* to solve each eGPR and identify minimal gene interventions (*Supplementary Figure 1D*). When Boolean network inconsistency occurs—defined as the condition where eGPR becomes unprogrammable (the target reaction becomes disabled)—the algorithm implements an adaptive strategy. This involves iterative reduction of regulatory layers until network consistency is achieved, ensuring robust problem solvability and biological relevance.

**
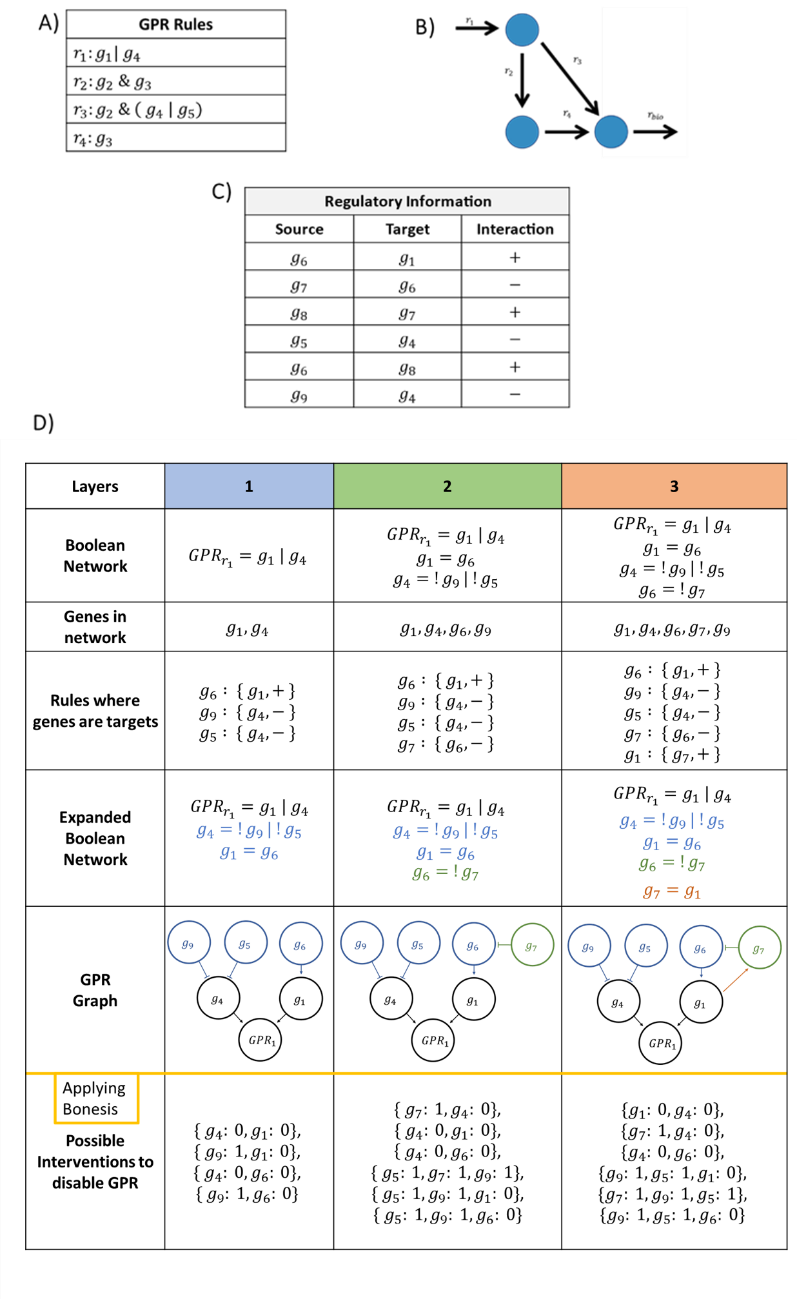
**

**Supplementary Figure 1. Integration of gene-protein rules with regulatory networks to construct layered Boolean models**. **(A)** Gene-protein rules (GPRs) defining a set of Boolean functions by which genes control protein assembly. Each rule (*r₁–r₄*) specifies the genetic requirements for protein synthesis using AND (&) and OR (|) operators. **(B)** Representation of the toy metabolic model associated with panel A. **(C)** Regulatory interaction network describing transcriptional control relationships between genes. Each interaction specifies a source and gene target gene, and the sign of interaction (+ for activation, − for repression). If multiple genes have the same target they are written in disjunctive form (OR). **(D)** Stepwise expansion of GPR of reaction 1 (GPR_r1_) across three network layers by incorporating regulatory information. Layer 0 contains the original metabolic GPR. Layer 1 integrates the genes in the GPR with their upstream regulators according to the interaction rules (blue). Layer 2 (green) further expands the network by recursively adding the genetic interactions as rules to the Boolean network. The expanded Boolean network row shows the complete regulatory logic after expansion with Layer 3 additions in orange. Finally, the resulting minimal gene intervention sets for reaction 1 and different layers using Bonesis. These intervention sets from all GPRs in the network collectively constitute GDict.

Once GDict is determined, the list of gMISs are computed using the MILP framework described above. As noted in the main text, gMISs are used to predict single genetic interventions (vulnerabilities) based on the gene expression levels of the rest of the genes, as introduced in Supplementary Figure 2. Specifically, following the approach presented in Barrena et al., 2025, we classify a gene knockout *g_i_^-^* as potentially essential in a particular sample if it is the unique highly expressed gene among the specific subset of gene knock-outs in a particular gMIS; however, it is also necessary to check that the subset of genes to be knocked-in in that gMIS are highly expressed. In addition, we can classify a gene knock-in *g_i_^+^* as a potential tumor suppressor gene if it is the unique lowly expressed gene among the specific subset of gene knock-ins in a particular gMIS; however, it is also necessary to check that the subset of genes to be knocked-out in that gMIS are lowly expressed.

To rigorously evaluate the lethality of single genetic interventions, we made use of Bonesis to assesses the propensity of eGPRs to undergo re-adaptation under realistic dynamic conditions. The validation procedure exhaustively tests all possible gene combinations within each gMIS using a leave-one-out strategy. Specifically, for a given gMIS, each possible gene intervention is forced in the Boolean network defined by its associated eGPRs and Bonesis check whether we can reach an attractor with the rest of the genes in the desired state: high expression for gene knock-ins and low expression for gene knockouts. Supplementary Figure 2 shows the following gMIS: {$g_{5}^{+},g_{6}^{-}, g_{9}^{+}$} and the analysis for the three possible single genetic interventions. First, Bonesis calculates whether an attractor exists with $g_{5}=g_{9}= 1$ (regulatory constraints) while forcing $g_{6}= 0$, which is possible in this case. Similarly, Bonesis searches for an attractor with $g_{6}=0, g_{9}= 1$ while forcing $g_{5}= 1$, infeasible under this setting (Supplementary Figure 2), since we reach an oscillatory attractor involving $g_{6}$ and, thus, $g_{6}=0$ is not guaranteed. A similar solution is found here for $g_{9}=1$ (Supplementary Figure 2).


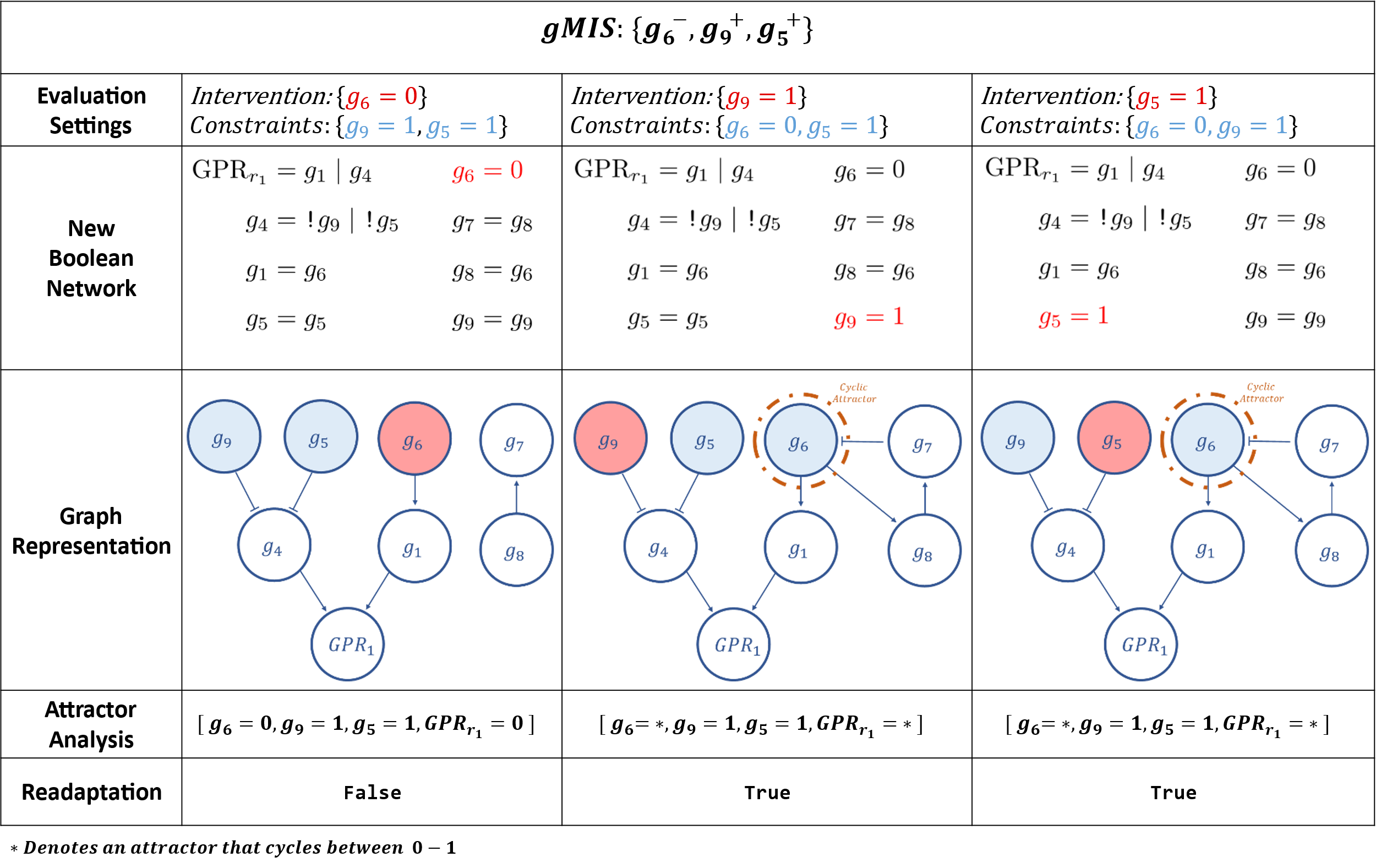
In summary, if the network converges to an attractor under the imposed constraints, the intervention does not trigger readaptation, indicating that the target intervention would be effective to achieve the desired objective. Conversely, the failure to reach an attractor in any configuration signifies that the network undergoes readaptation, deploying compensatory mechanisms that could circumvent the intended genetic intervention. This validation approach addresses a critical limitation of purely stoichiometric optimization methods, specifically the assumption of steady-state conditions without consideration of regulatory dynamics. Networks exhibiting cyclic attractors or unstable dynamics under intervention conditions reveal potential mechanisms of therapeutic resistance or metabolic compensation that would not be apparent from constraint-based modeling exclusively.

**Supplementary Figure 2. Post-processing validation of single genetic interventions via Bonesis.** Computational workflow for evaluating single genetic intervention stability. Each identified gMIS is analyzed with the eGPRs of its associated genes. A toy example is shown for the gMIS: {$g_{5}^{+},g_{6}^{-}, g_{9}^{+}\}$ and different single interventions. First, Bonesis assesses whether a stable attractor can be reached with g*_5_* = g*_9_* = 1 by forcing g*_6_* = 0, which is possible here. In addition, we analyze the cases of g*_6_* = 1 and g*_9_* = 1, where the validation fails because g₆ enters a cyclic attractor (indicated by *), demonstrating that the regulatory network possesses mechanisms to circumvent the intervention through oscillatory dynamics.


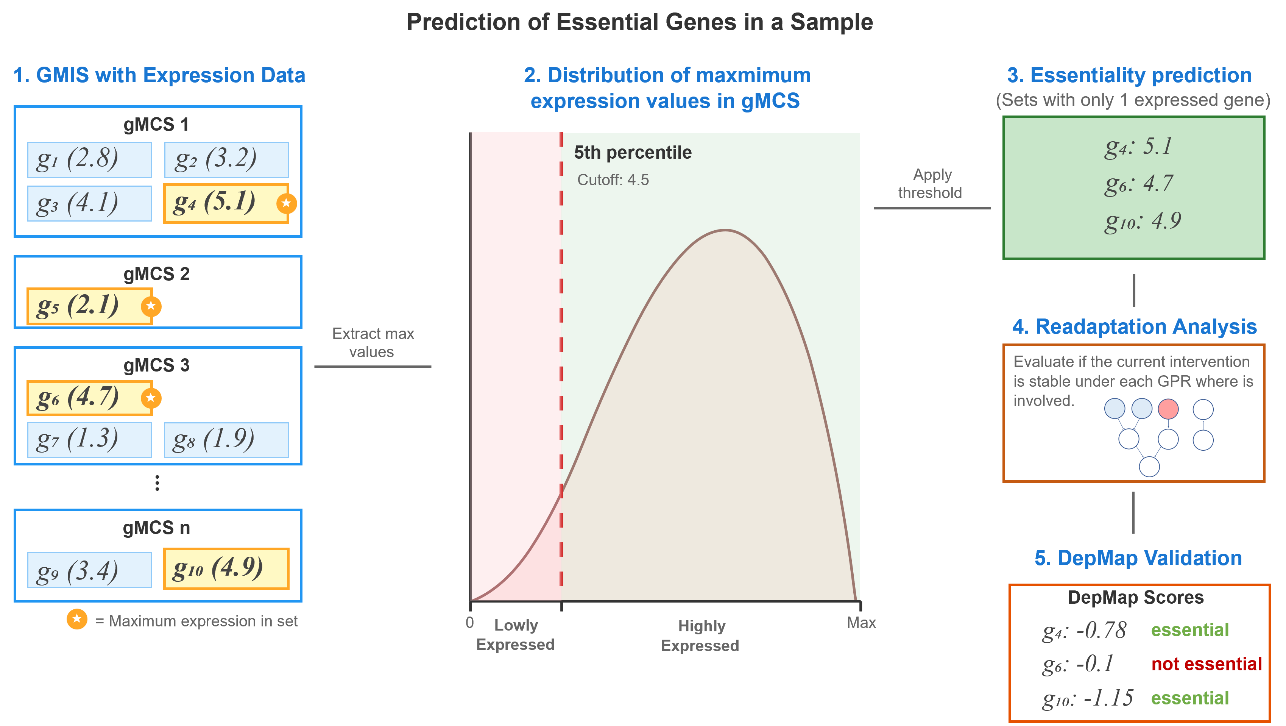
**Supplementary Figure 3: gMIS-based gene essentiality analysis.** For each sample in the DepMap database (Arafeh et al, 2025), we evaluated the expression levels of the genes involved in our list of genetic Minimal Intervention Sets (gMIS). In order to discretize expression levels into highly and lowly expressed genes, we used the gmcsTH5 approach, introduced in Valcarcel et al. 2024, briefly summarized below. The maximum expression value from each gMIS was extracted, with duplicate gene-expression pairs removed to ensure independence (1). These maximum values were aggregated to construct an empirical distribution, from which we calculated a sample-specific expression threshold, gmcsTH5, defined as the 5th percentile (2). While this percentile cutoff is arbitrary, it was selected to identify genes with markedly low expression relative to the gMIS expression landscape of the sample. (3) Using gmcsTH5 as a classifier, genes were categorized as either expressed (≥ gmcsTH5) or lowly expressed (< gmcsTH5). We subsequently filtered for gMIS containing exactly one expressed gene, as such sets represent predicted essentialities—the single expressed gene should be indispensable for cellular viability given the constraint that at least one gene from each set must be expressed. (4) The re-adaptation analysis discussed above is applied and those essential genes that do not satisfy the desired constraints are discarded. (5) To validate our predictions, we compared them against experimental knockout data from the DepMap CRISPR screen database. Genes with knockout fitness scores ≤ −0.6 were classified as experimentally essential. This comparison enabled the calculation of standard classification metrics: true positives (TP) when a predicted essential gene showed knockout fitness ≤ −0.6; false positives (FP) when predicted essentials were not essential by knockout data; false negatives (FN) when experimentally essential genes were not predicted as essential; and true negatives (TN) for all remaining genes. This framework allows systematic evaluation of expression-based essentiality predictions across diverse cellular contexts.

**
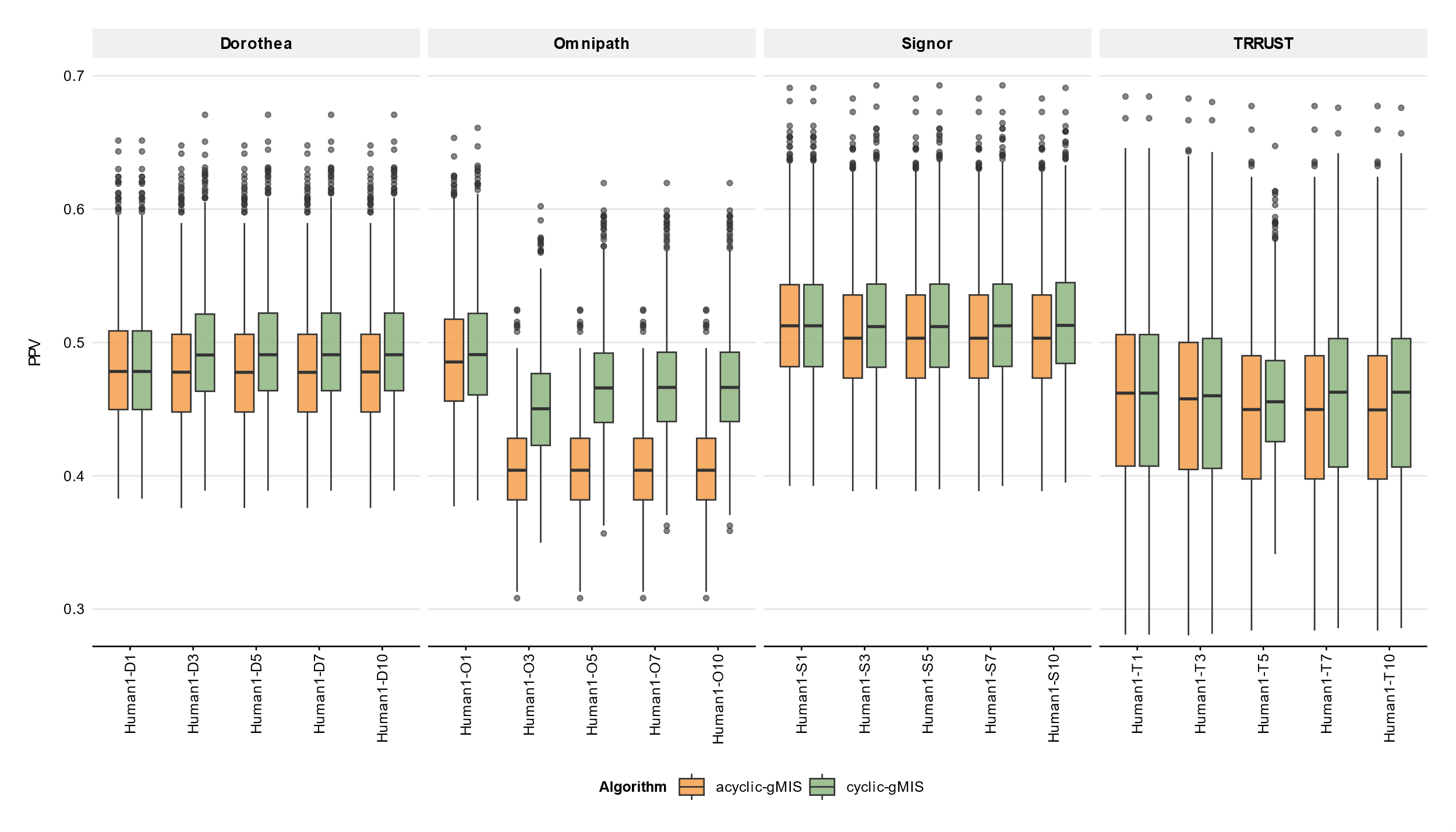
Supplementary Figure 4: Positive Predictive Value comparison across network configurations and algorithms.** Positive Predictive Value (PPV) grouped by network configuration and algorithm. Network configurations include GEM Humanv1.19 combined with multiple networks: Dorothea, Omnipath, Signor, and TRRUST. Each configuration is subdivided into two groups: acyclic gMIS and cyclic gMIS, with facets according to the number of layers used in the analysis (1,3,5,7,10).


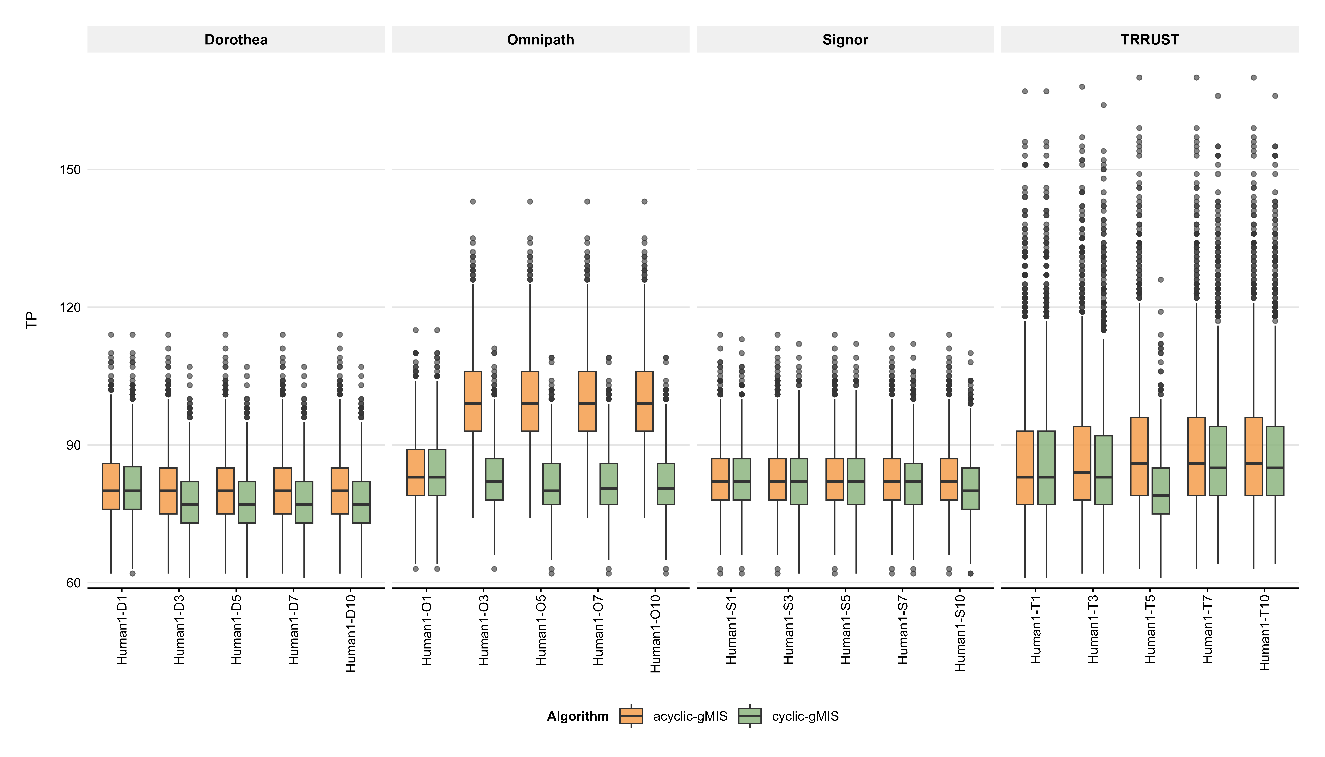
**Supplementary Figure 5: True Positive comparison across network configurations and algorithms.** True Positives (TP) grouped by network configuration and algorithm. Network configurations include GEM Humanv1.19 combined with multiple networks: Dorothea, Omnipath, Signor, and TRRUST. Each configuration is subdivided into two groups: acyclic gMIS and cyclic gMIS, with facets according to the number of layers used in the analysis (1,3,5,7,10).


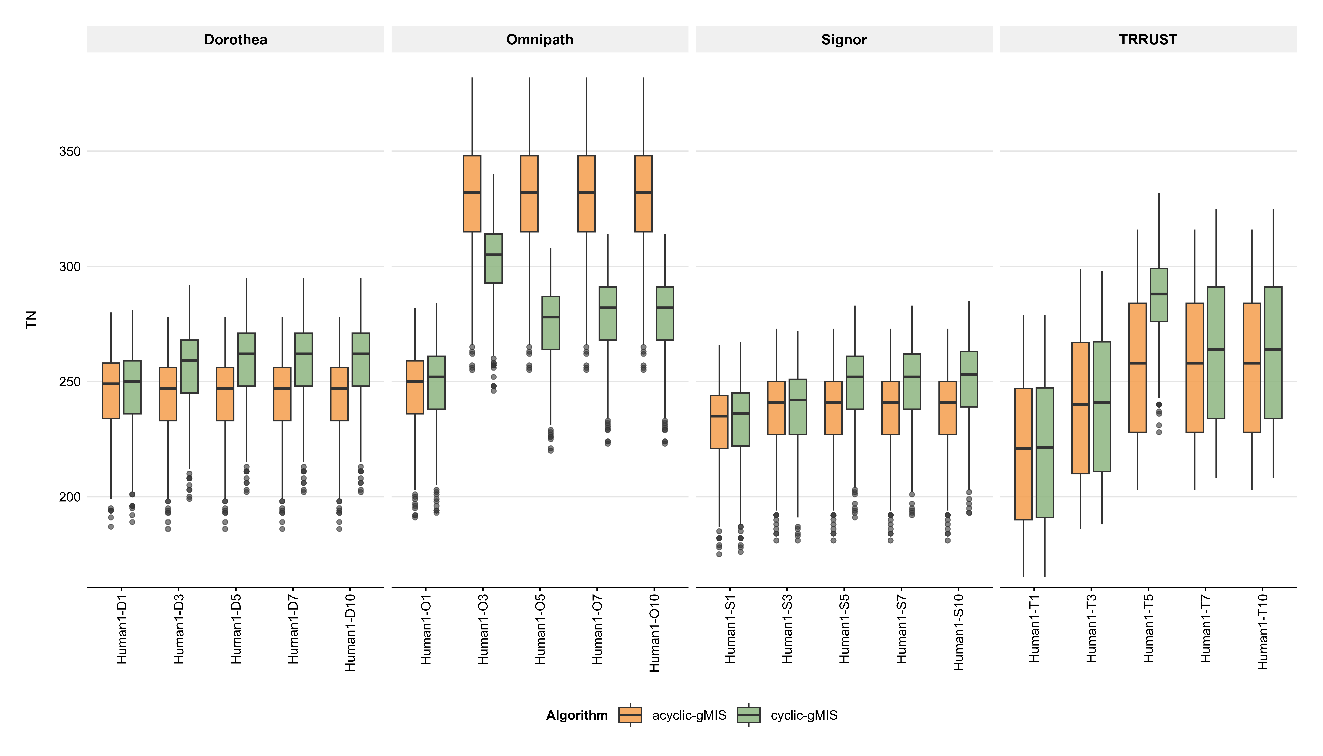

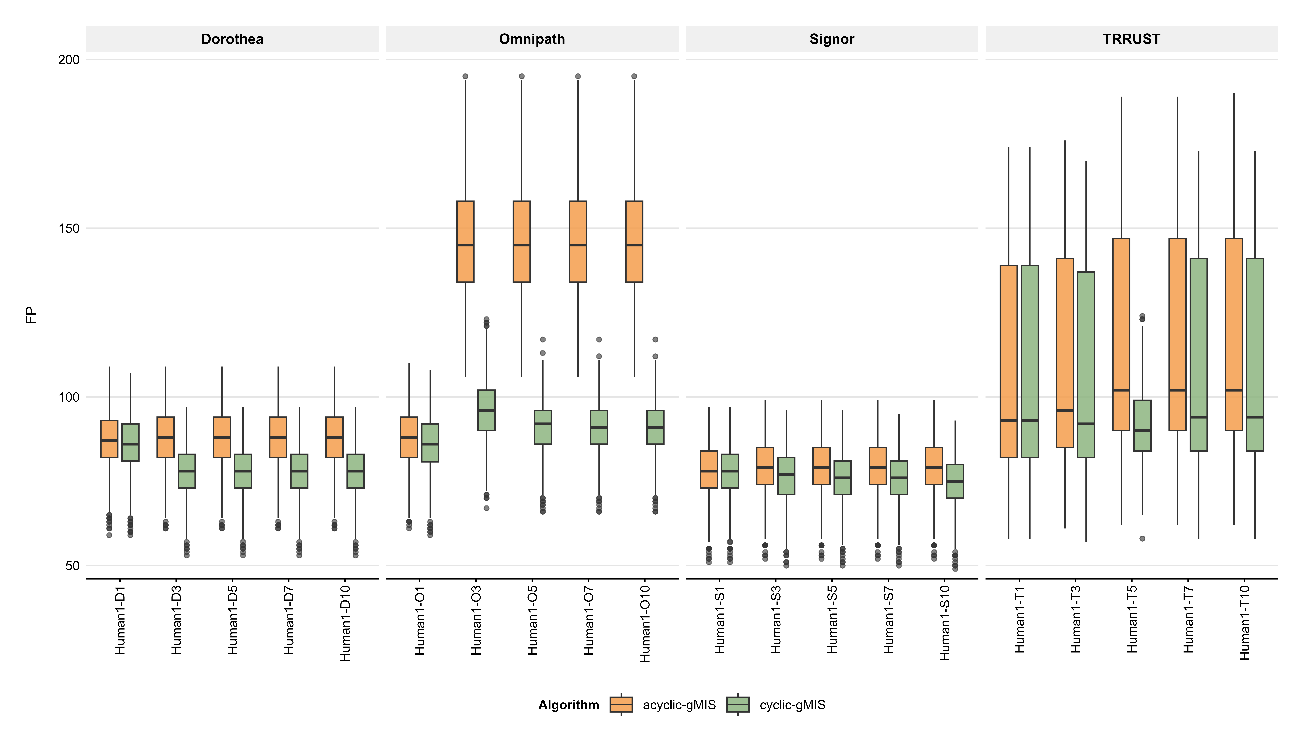
**Supplementary Figure 6: False Positive comparison across network configurations and algorithms.** False Positives (FP) grouped by network configuration and algorithm. Network configurations include GEM Humanv1.19 combined with multiple networks: Dorothea, Omnipath, Signor, and TRRUST. Each configuration is subdivided into two groups: acyclic gMIS and cyclic gMIS, with facets according to the number of layers used in the analysis (1,3,5,7,10).

**Supplementary Figure 7: True Negative comparison across network configurations and algorithms.** True Negatives (TN) grouped by network configuration and algorithm. Network configurations include GEM Humanv1.19 combined with multiple networks: Dorothea, Omnipath, Signor, and TRRUST. Each configuration is subdivided into two groups: acyclic gMIS and cyclic gMIS, with facets according to the number of layers used in the analysis (1,3,5,7,10).

**Supplementary Figure 8: False Negative comparison across network configurations and algorithms.** False Negative (FN) grouped by network configuration and algorithm. Network configurations include
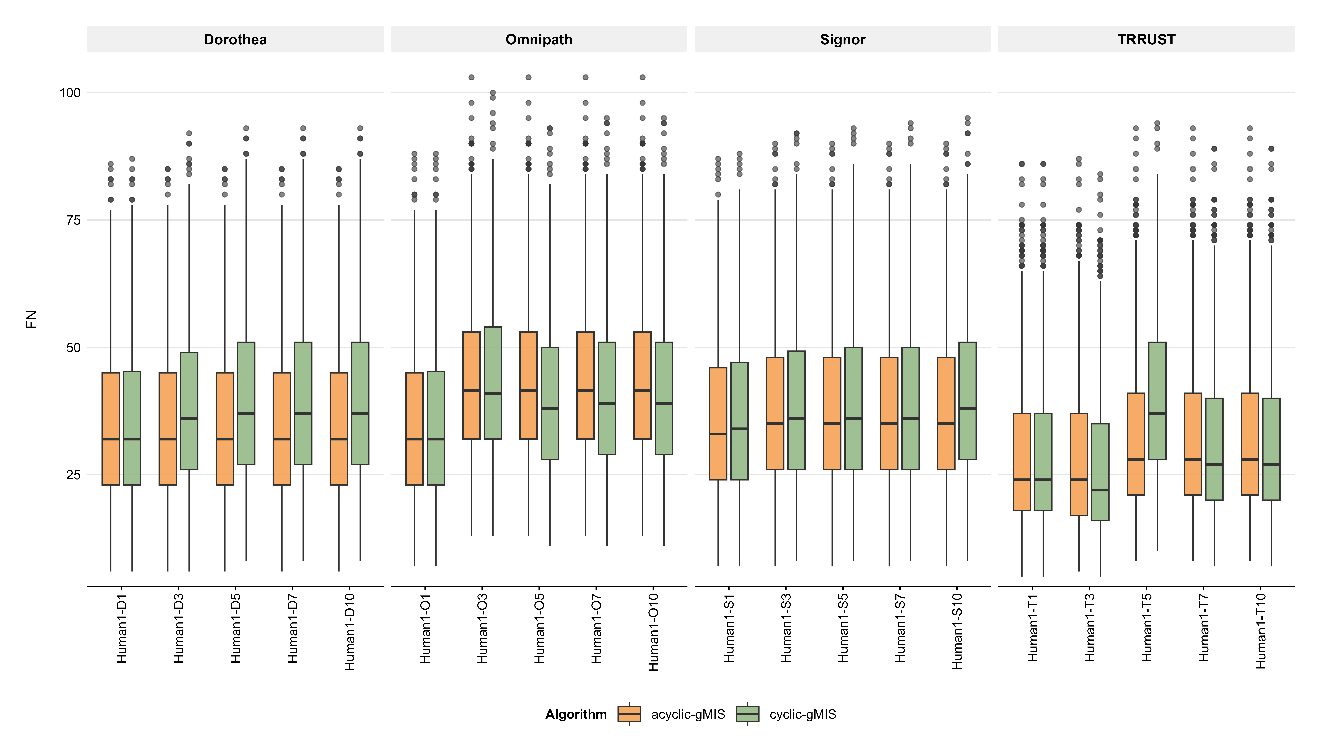
GEM Humanv1.19 combined with multiple networks: Dorothea, Omnipath, Signor, and TRRUST. Each configuration is subdivided into two groups: acyclic gMIS and cyclic gMIS, with facets according to the number of layers used in the analysis (1,3,5,7,10).


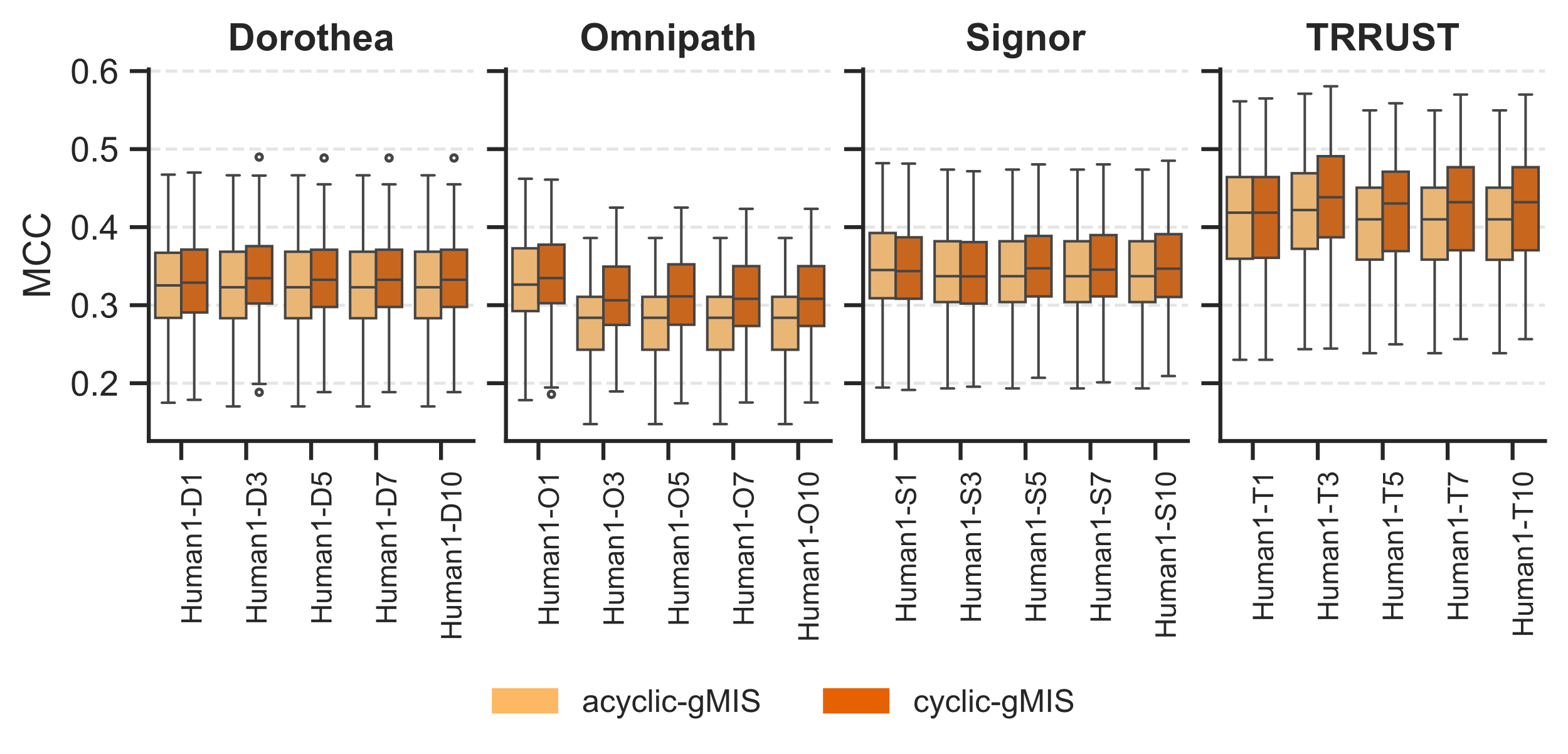
**Supplementary Figure 9:** **Benchmark against Project Score data**. Matthews Correlation Coefficient (MCC), grouped by network configuration and algorithm, based on Project Score data. The workflow used to benchmark against Project Score data consisted of projecting the sequenced data of the 580 cell lines in the library and using the binary dependency matrix to evaluate whether the predicted essential gene was a dependency in the experimental data. Network configurations include GEM Humanv1.19 combined with multiple regulatory networks: Dorothea, OmniPath, Signor, and TRRUST. Each configuration is subdivided into two groups — acyclic gMIS and cyclic gMIS — with facets according to the number of layers used in the analysis (1, 3, 5, 7, 10).

**
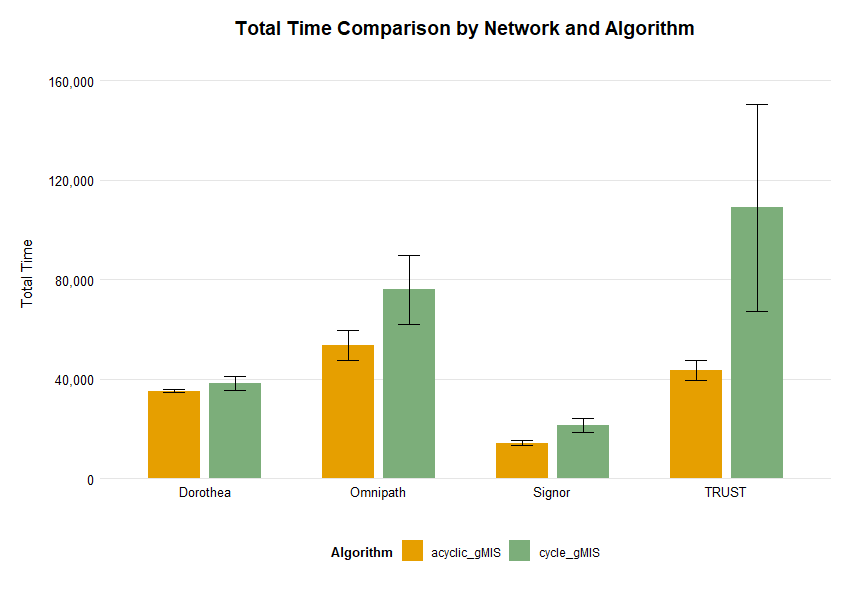
**

**Supplementary Figure 10: Computation time comparison across network configurations and algorithms.** A bar chart comparing the total computational time (in seconds) for the *acyclic-gMIS* (yellow) and *cyclic-gMIS* (green) algorithms across four regulatory databases, with error bars indicating standard deviation, using standardized computational resources (16 threads, 32GB RAM). The *cyclic-gMIS* algorithm consistently incurs a higher computational cost than its acyclic counterpart. This increased runtime, which is most pronounced for the TRRUST network, represents an expected trade-off for the added complexity of processing and validating cyclic regulatory structures.


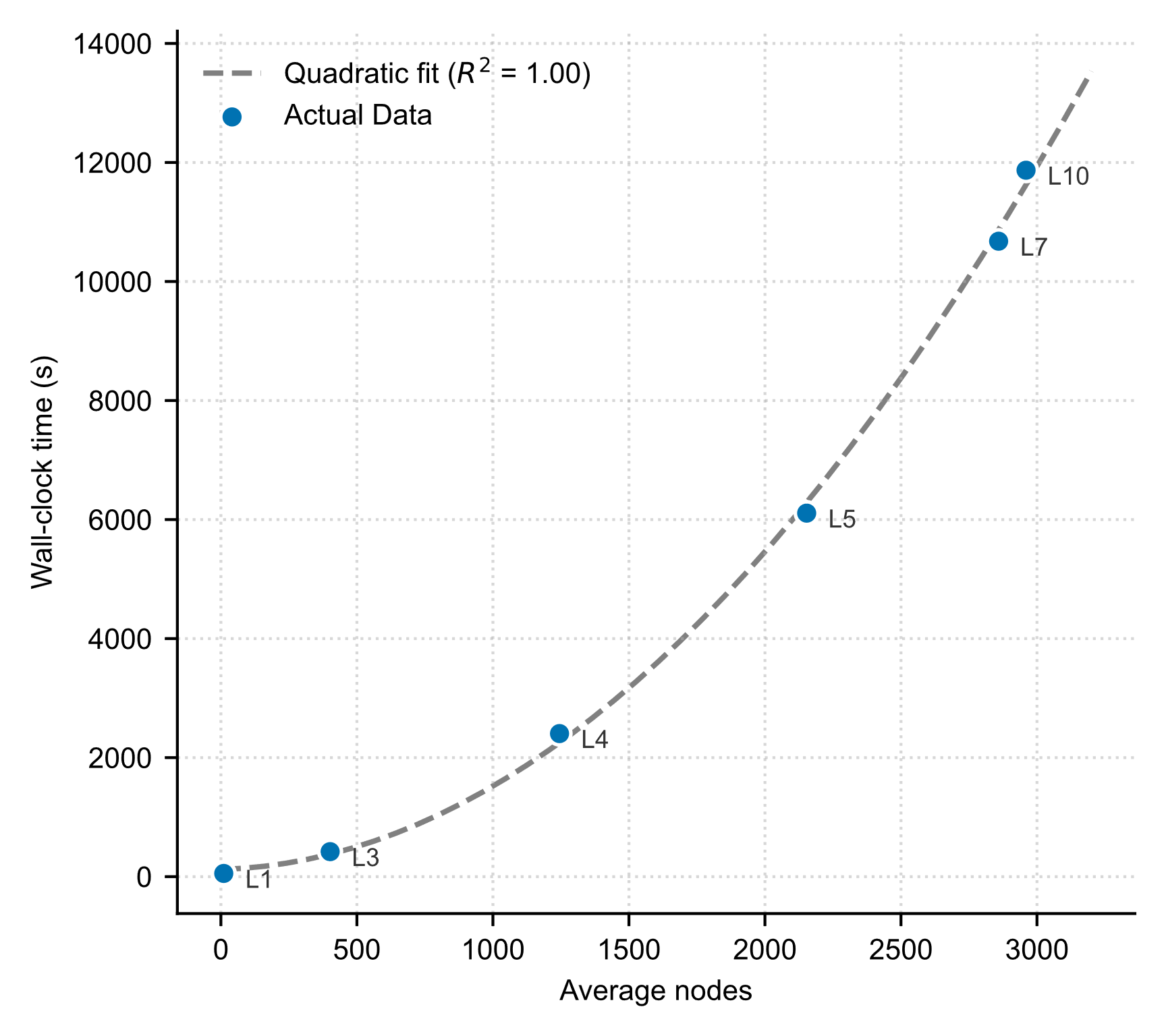


**Supplementary Figure 11: Regression analysis of the readaptation cost across the different layers of OmniPath.** We measured the wall-clock time for attractor-based readaptation method across the largest network (OmniPath) with varying layer depths, using standardized computational resources (16 threads, 32GB RAM) for each run. The computational time scales quadratically with the number of nodes (R² = 1.00). To evaluate worst-case scenarios, we extrapolated this relationship to predict performance for networks with 6,000 nodes—representing approximately double the average node count observed in OmniPath. Under these extreme conditions, a network with 6,000 nodes per GPR would require approximately 50,000 seconds (~14 hours) to complete the analysis of all GPRs. Although attractors are also employed when calculating intervention strategies for each GPR, this calculation phase is mixed with MILP calculations for GPRs lacking regulatory information; therefore, to more accurately measure the impact of attractors, we chose the method that relies more heavily on them.

**
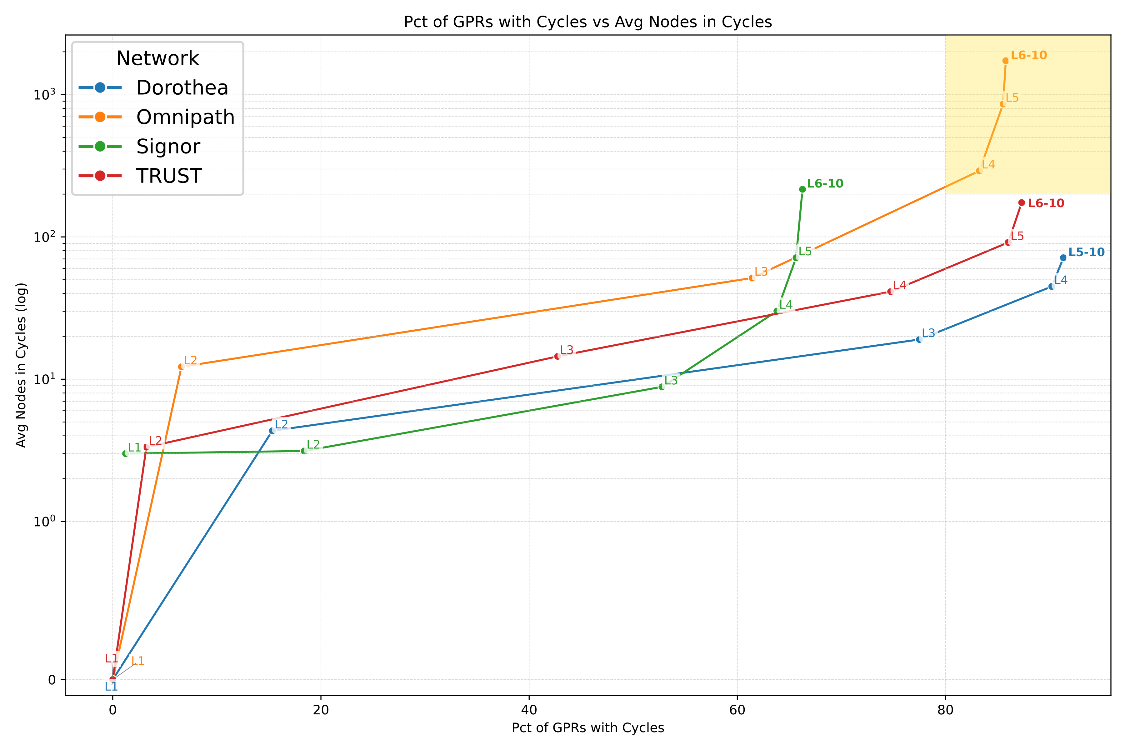
S****upplementary Figure 12: Relationship between percentage of GPRs with cycles and the average nodes in cycles.** Each point corresponds to a regulatory layer in a specific model (layer X is denoted as LX or group of layers X-Y as LX-Y). We analyzed the generated eGPRs for each different integrated model. We measured the percentage of eGPRs with at least one cycle and the average number of nodes involved in cycles per GPR. Omnipath shows a higher cycle burden at deeper regulatory layers than the other models, reaching thousands of nodes in the deeper layers (L6-10).


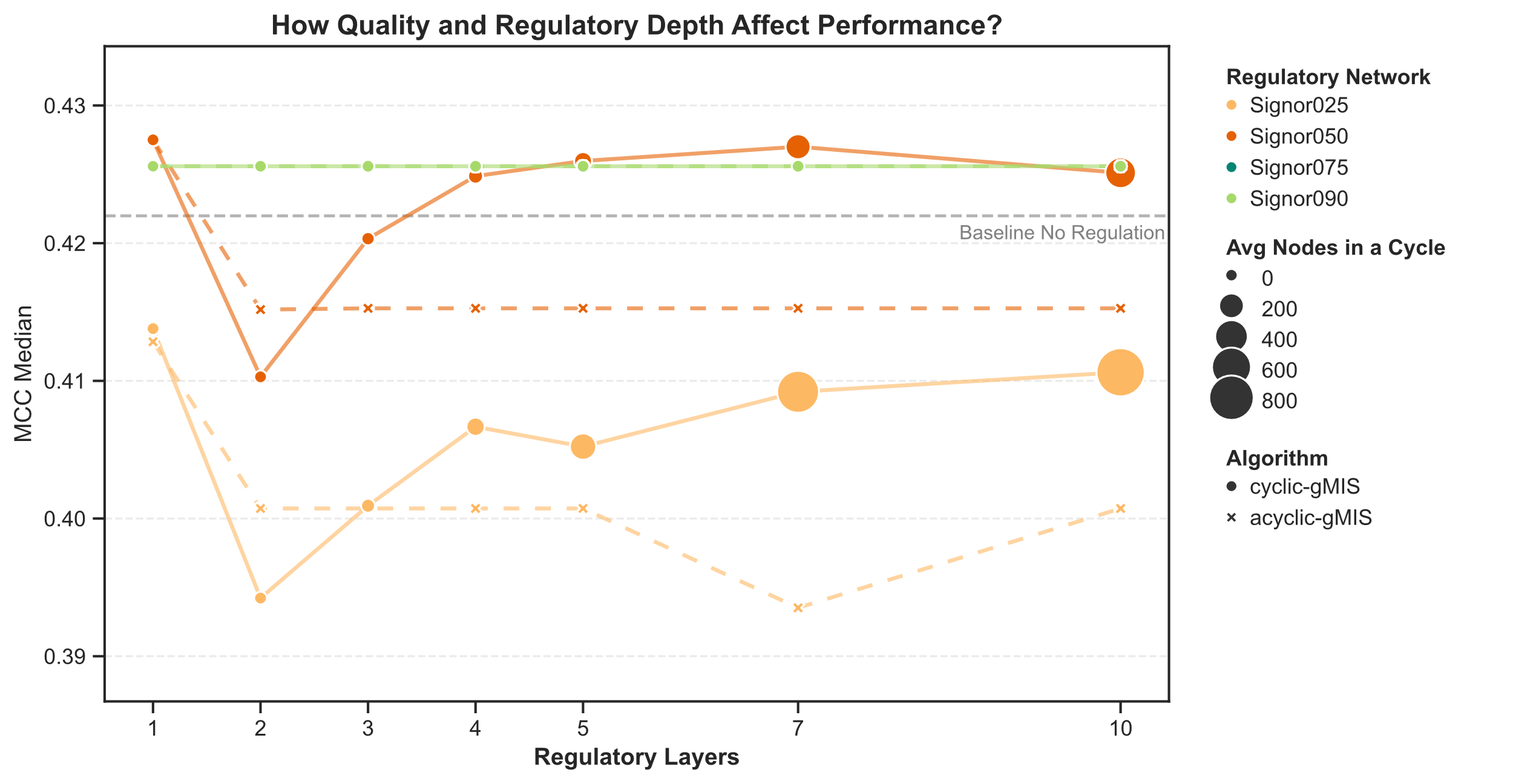


**Supplementary Figure 13: SIGNOR networks quality versus networks coverage.** Using the SIGNOR regulatory network, we created three versions with increasing confidence thresholds (0.25, 0.50, 0.90), which naturally produces networks of decreasing coverage of GPRs (n=337, 163 and 22 affected GPRs, respectively) but increasing interaction quality. As network quality increases the number of genes participating in cycles decrease, network size decreases as well as GPR coverage. We included both algorithms in the benchmark to asses how networks with higher quality would behave as regulatory depth increases. To further illustrate the scale of dimensionality reduction across confidence thresholds, Signor025 contains 10,452 regulatory interaction pairs, Signor050 contains 4,810, and Signor090 contains 304.

| **Network** | **acyclic-GmispY Mean** | **CycLIC gmispY Mean** | **Gain (Δ)** | **P-value** | **% Gain** |
| --- | --- | --- | --- | --- | --- |
| **Human1-Omnipath** | 0.288 | 0.316 | 0.028 | <0.001 | **9.74** |
| **Human1-Dorothea** | 0.324 | 0.333 | 0.009 | <0.001 | **2.77** |
| **Human1-Signor** | 0.339 | 0.347 | 0.007 | <0.001 | **2.13** |
| **Human1-TRRUST** | 0.412 | 0.423 | 0.011 | <0.001 | **2.68** |

**Supplementary Table *1*: Summary of Project Score comparison between gMISpy-acyclic and gMISpy-cyclic**. Mean MCC value across samples for gMISpy-acyclic and gMISpy-cyclic, their absolute and relative gain, t-test p-values.

**References**

Apaolaza, I., San José-Eneriz, E., Tobalina, L., Miranda, E., Garate, L., Agirre, X., Prósper, F., & Planes, F. J. (2017). An in-silico approach to predict and exploit synthetic lethality in cancer metabolism. *Nature Communications 2017 8:1*, *8*(1), 1–9. https://doi.org/10.1038/s41467-017-00555-y

Apaolaza, I., Valcarcel, L. V., & Planes, F. J. (2019). GMCS: Fast computation of genetic minimal cut sets in large networks. *Bioinformatics*, *35*(3), 535–537. <https://doi.org/10.1093/bioinformatics/bty656>

Arafeh, R., Shibue, T., Dempster, J.M.et al.The present and future of the Cancer Dependency Map.Nat Rev Cancer 25, 59-73 (2025). https://doi.org/10.1038/s41568-024-00763-x

Barrena, N., Valcárcel, L. V, Olaverri-Mendizabal, D., Apaolaza, I., & Planes, F. J. (2023). Synthetic lethality in large-scale integrated metabolic and regulatory network models of human cells. *Npj Systems Biology and Applications*, *9*(1), 32. https://doi.org/10.1038/s41540-023-00296-3

Valcárcel, L.V., San José-Enériz, E., Ordoñez, R. et al. An automated network-based tool to search for metabolic vulnerabilities in cancer. Nat Commun 15, 8685 (2024). https://doi.org/10.1038/s41467-024-52725-4
